# Zebrafish as an Emerging Model for Osteoporosis: A Primary Testing Platform for Screening New Osteo-Active Compounds

**DOI:** 10.3389/fendo.2019.00006

**Published:** 2019-01-29

**Authors:** Dylan J. M. Bergen, Erika Kague, Chrissy L. Hammond

**Affiliations:** ^1^School of Physiology, Pharmacology and Neuroscience, Biomedical Sciences Building, University of Bristol, Bristol, United Kingdom; ^2^Musculoskeletal Research Unit, Translational Health Sciences, Bristol Medical School, Southmead Hospital, University of Bristol, Bristol, United Kingdom

**Keywords:** zebrafish, screening, genetic mutants, osteoblast, osteoclast, osteoporosis, drug development, animal model

## Abstract

Osteoporosis is metabolic bone disease caused by an altered balance between bone anabolism and catabolism. This dysregulated balance is responsible for fragile bones that fracture easily after minor falls. With an aging population, the incidence is rising and as yet pharmaceutical options to restore this imbalance is limited, especially stimulating osteoblast bone-building activity. Excitingly, output from large genetic studies on people with high bone mass (HBM) cases and genome wide association studies (GWAS) on the population, yielded new insights into pathways containing osteo-anabolic players that have potential for drug target development. However, a bottleneck in development of new treatments targeting these putative osteo-anabolic genes is the lack of animal models for rapid and affordable testing to generate functional data and that simultaneously can be used as a compound testing platform. Zebrafish, a small teleost fish, are increasingly used in functional genomics and drug screening assays which resulted in new treatments in the clinic for other diseases. In this review we outline the zebrafish as a powerful model for osteoporosis research to validate potential therapeutic candidates, describe the tools and assays that can be used to study bone homeostasis, and affordable (semi-)high-throughput compound testing.

## Introduction

Osteoporosis (OP) is a degenerative bone disease that affects around 27.6 million people over the age of 50 in the 27 European Union (EU27) countries alone (1). As average life expectancies increase, it is predicted that the annual cost of treating OP in the EU will rise from €37 billion in 2010 to €46.5 billion by 2025 (2). OP is characterized by a reduction in bone mineral density (BMD), reduction of bone mass (BM), and a decrease in the trabecular volume of long bones; resulting in brittle bones that are more prone to fracture (3). The underlying mechanism behind OP is a dysregulation of bone homeostasis; with decreased bone anabolism (decreased activity of osteoblasts and osteocytes) and increased catabolism (enhanced osteoclast activity). Successful treatment of OP should therefore increase bone anabolism and decrease catabolism to reinstate the equilibrium in bone homeostasis (4, 5). While therapeutic options are increasing, all but one available therapies aim to reduce bone resorption. However, as osteoclast and osteoblast activity are coupled, anti-resorptives can negatively affect anabolic osteoblast activity and may not fully restore bone architecture (6). The only injectable osteoanabolic compound, teriparatide, is an analog of the parathyroid hormone (7). However, it is not an ideal long-term therapy option as, not only is it expensive, long term exposures in rat increase susceptibility to osteosarcoma (8, 9) limiting treatment duration (currently 2-years) in OP patients (10). Thus, an ideal treatment plan should focus on both strengthening bones using an osteoanabolic compound, combined with use of an anti-resorptive treatment (also ideally non-invasive) to maintain bone integrity (5), few such options exist. Currently, a major bottleneck in the development of new pharmaceuticals is the collection of primary functional data on new biological drug targets with osteo-anabolic capacities.

The twinning of genetic information with mechanistic data is key for development of new treatments. For example, familial studies on high bone mass (HBM) cases led to the discovery of mutations in *SOST* (*Sclerostin*). Further mechanistic data generated in model systems showed that SOST acts negatively on the WNT signaling pathway and led to the development of a novel antibody treatment Romosozumab (approved in 2018 for clinical use), which blocks SOST activity (11–13). With the advent of genome-wide association studies (GWAS), and efficient whole-genome/exome sequencing (WGS/WES) data mapping there has been a sizeable increase in availability of human genetic data from cohort studies for musculoskeletal conditions including OP, high bone mass (HBM), and osteoarthritis (OA) (14–20). Recent large cohort studies, such as UK-Biobank, have identified many new loci that contain novel osteogenic factors. For example, the UK-Biobank (21) data yielded 518 loci associated with changes in BMD using heel ultrasound data (16, 19). Currently, there is a substantial gap in translating these human genetic findings to model systems (22) in which the mechanism by which these genes act on the skeleton can be defined, where hypotheses can be tested, and ultimately define new putative drug targets that can be assessed with pharmacological agents. Because the skeletal system involves complex interactions between different cell and tissue types, genes and mechanical stimuli it is difficult to recapitulate features of OP in a petri dish. However, traditional rodent models are expensive to genetically manipulate. Zebrafish (*Danio rerio*) could therefore bridge this gap by offering fast genetic manipulation and complex tissue interactions required to model complex diseases such as OP.

Zebrafish are vertebrates and show strong similarities in their skeletal physiology to mammals (23). They are highly fecund and a single pair of fish can lay up to 300 eggs a week, which develop externally and are translucent (24). They show conservation of 70% of all genes and 85% of disease genes with humans (25, 26). However, the main advantage of zebrafish for functional genetic studies is their genetic tractability, as constructs that modify the genome can be injected directly into embryos at the single cell stage. This has allowed the generation of transgenic lines that allow dynamic imaging of all the cells of the developing skeletal system in live larvae (27–29) (Table 1) and in more recent years allowed genome editing strategies to be employed. In this review we set-out these different approaches and how developing and adult zebrafish can be used to study bone mineralization, bone content formation, and osteoblast-osteoclast interactions in a whole animal context. We also discuss future prospects for drug screening pipelines in zebrafish which may confer advantages over other pre-clinical model systems.

**Table 1:** Common transgenic lines to study musculoskeletal system in small teleostei.

**Gene/pathway**	**Cell type(s)**	**Description**	**Transgenic line**	**Citation**
*BMP pathway*	BMP transcriptionally activated cells	Reporter—21 BMP responsive elements (BMPRE) from *X. laevis*	*Tg(5xBMPRE-Xla.Id3:GFP)*	(30)
*collagen10a1a*	Osteoblasts (juvenile)	Reporter—BAC containing zebrafish collagen10a1a promoter	*TgBAC(col10a1a:Citrine)*	(29)
*collagen2a1*	Chondrocytes	Reporter—BAC containing zebrafish *collagen2a1* promoter	*Tg(Col2a1aBAC:mCherry)*	(29)
*ctsk*	Osteoclasts	Reporter—BAC containing zebrafish *ctsk* promoter	*TgBAC(ctsk:Citrine)*	(27)
*entpd5a*	Mineralizing osteoblasts	Reporter—BAC containing zebrafish *entpd5a* promoter	*TgBAC(entpd5a:Citrine/YFP)*	(27)
*fli1a*	Vasculature/neural crest	Reporter —BAC containing *fli1a* promoter	*Tg(fli1a:EGFP)*	(31)
*Hedgehog pathway*	Gli transcriptionally activated cells	Reporter—8 Gli responsive elements driving *egfp* or *mCherry*	*Tg(Gli-d:gfp/mCherry)*	(32)
*Osteocalcin*	Osteoblasts (mature)	Reporter—3.7 kb upstream osteocalcin promoter from Medaka driving *gfp* expression	*Tg(Ola.osteocalcin:EGFP)*	(33)
*rankl*	Osteoclast-osteoblast interaction	Conditional—Heat shock inducible (HSE) ubiquitous simultaneous expression of *rankl* and *cfp* in medaka	*Tg(rankl:HSE:CFP)*	(34)
*runx2*	Osteoblasts (juvenile) forming new bone	Reporter—557 bp intronic human *RUNX2* enhancer (Hsa), regulating *RUNX2*, conserved in multiple species, driving *gfp* expression	*Tg(Hsa.RUNX2-Mmu.Fos:EGFP)*	(33)
*sox10*	Mesenchymal chondrocytes	Reporter—4.9 kb of *sox10* promoter driving *egfp*	*Tg(−4.9Sox10:EGFP)*	(35)
*sp7 (osx)*	Osteoblasts	Reporter—BAC containing zebrafish *sp7* promoter	*Tg(sp7:EGFP)*	(36)
*sp7 (osx)*	Osteoblasts	Reporter—Medaka *sp7* regulatory elements driving *nls-gfp* or *mCherry*	*Tg(sp7:nuGFP/mCherry) or Tg(Ola.sp7:NLS-GFP)*	(37)
*sp7 (osx)*	Osteoblasts	Reporter—BAC *sp7* promoter driving *luciferase* expression	*Tg(Ola.sp7:luciferase)*	(38)
*sp7 (osx)*	Osteoblasts (ablation)	Conditional—Chemical ablation of osteoblasts by *E. coli* enzyme Nitroreductase (NTRo) activity	*Tg(osterix:mCherry-NTRo)pd46*	(39)
*WNT - β-catenin pathway*	β-catenin activated cells	Reporter—T-cell factor enhancer (TCF) promoter containing 7 beta-catenin binding sites	*Tg(7xTCF.XlaSiam:nlsGFP)*	(40)

*BAC, bacterial artificial chromosome; bp, base pair; kb, kilobase*.

## Flexible Genetic Manipulation in the Zebrafish

Zebrafish are genetically high amenable and new ways to manipulate the genome are constantly being added to the zebrafish genetic toolbox, which includes knockout, knock-down and, DNA insertion strategies. The external development of the embryos allows tools targeting genes of interest to be microinjected directly in embryos at the 1-cell stage and hundreds of embryos can readily be injected in a morning. Acute knockdown of gene expression can be achieved either by targeting mRNA with antisense RNA morpholino (MO) molecules that stably bind the target mRNA to block translation or splicing through steric hindrance (41). MOs offer a rapid method to assess the phenotype of a gene of interest during early development. However, they can only be used to study developmental processes occurring over the first 4 or 5 days of development, which limits their utility in skeletal studies as mineralization occurs from 4 days of development. While concerns have been raised about MO veracity as morphants frequently show more severe phenotypes than stables mutants generated for the same gene (42, 43). This is due to a transcriptional compensation response for chronic loss of a gene as has been shown in mouse, cultured human cell lines, plants, and zebrafish models (44–51). Thus, while MOs have a role, their use has been largely supplanted by use of genome editing strategies.

**Figure 1:**
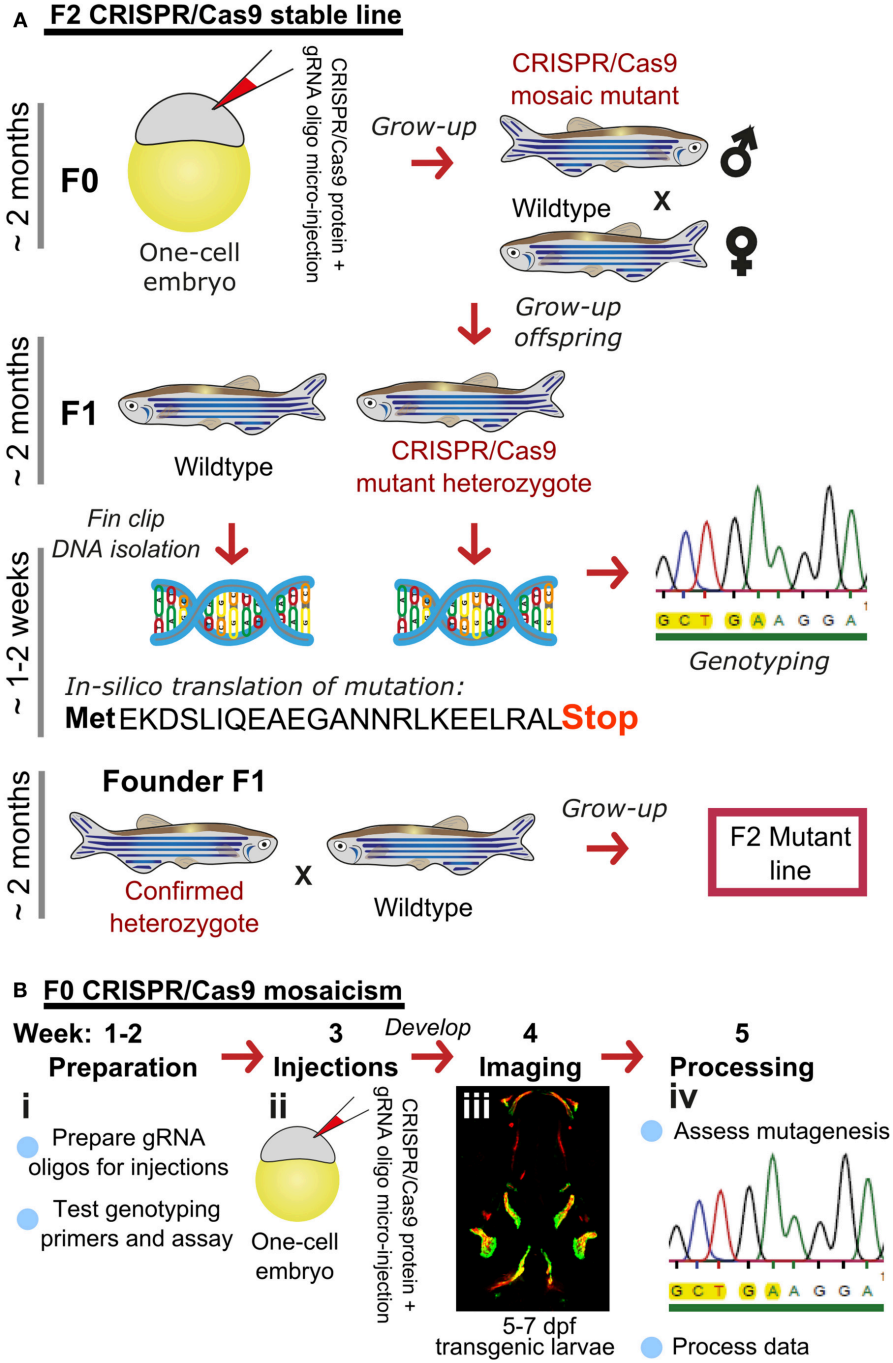
Rapid and efficient mutagenesis using CRISPR/Cas9 genome editing in zebrafish. **(A)** To generate a stable mutant line, F0 CRISPR/Cas9 injected individuals carrying mosaic mutations (defined by fin-clipping, **B**) should be outcrossed to wildtype fish to allow selection of a single germline mutation. Out-crossing the founder to wildtype will establish a stable F2 mutant line. Note that the F1 can have multiple founders with damaging mutations, incrossing these will result in F2 homozygotes (for recessive alleles) for functional analysis. When performing incrosses from F2, it will take another 2 months of breeding time. **(B)** This rapid protocol can be used to generate mutations in a gene of interest using CRISPR/Cas9 RNA or protein with gRNAs targeted against the gene from custom made gRNA oligos (i). Micro-injection of CRISPR/Cas9 RNA or protein and gRNAs specific to gene of interest into embryos at the single cell stage (ii) generating double stranded breaks during the first few rounds of cell divisions. The repair machinery is prone to errors and those cells will carry a different type of mutation giving a range of insertion and deletion (indel) mutations (spectrum of mutations, mosaicism). The overall mutagenic efficiency is typically high (around 80% with fragment analysis) allowing larval skeletal phenotypes to be assessed in the injected (F0) population (60). After imaging an Alizarin Red S (AR) stained individual in a transgenic background (here osteoblast marker *sp7:gfp*)(iii), mutagenesis assessment such as fragment analysis will determine a quantified mutagenesis rate (61) which can be correlated to a phenotype (iv). Note that mosaic mutants (crispants) can also be grown up to see the effect on the adult skeleton.

Traditionally, zebrafish mutant lines have been generated by forward genetic screening; using mutagens [e.g., N-ethyl-N-nitroso urea (ENU)] to induce random point mutations in offspring that were then screened for phenotypes of interest (52–56). The expansion of the zebrafish genetic toolkit with zinc-finger nucleases (ZFN), Transcription Activator-Like Effector Nucleases (TALEN) (57, 58), and Clustered Regularly Interspaced Short Palindromic Repeats (CRISPR)/Cas9 (59) reverse genetic strategies, which, in combination with a fully sequenced genome (25), allow tailored gene-specific mutagenesis in the zebrafish. Gene function can be studied in genetic knockouts by generating insertion/deletion (indel) mutations leading to premature stop codons, deleting whole exons containing important protein domains and generate new stable mutant lines (Figure 1A). Moreover, the CRISPR/Cas9 protocol is so efficient that the F0 injected fish (crispants) can be used to study loss of gene function in these crispants, despite them carrying mosaic mutations (i.e., not every cell carries a mutation and more than one mutation may be present) (23, 60) (Figure 1B). Single base gene editing (knock ins) using modified Cas9 enzymes or supplying a DNA template for the endogenous homologous recombination machinery initiated by a double stranded break allows to introduce specific genetic changes to model specific human disease mutations in zebrafish orthologs (62, 63).

## Simple Assessment of Zebrafish Bones During Development and Adulthood

Zebrafish in common with higher vertebrates, have both dermal/intramembranous ossification, in which bone is formed *de novo* directly by osteoblasts, and chondral/endochondral ossification in which bone forms by progressively replacing a cartilaginous template. Although zebrafish have thinner bones than terrestrial vertebrates, with fewer embedded osteocytes and little trabeculation, all of the relevant skeletal cell types and modes of regulation are conserved between zebrafish and higher vertebrates. This, importantly for the study of OP, includes osteoblast and osteoclast coupling and regulation of bone remodeling (64, 65).

A major advantage of using zebrafish to probe the mechanism of bone homeostasis is that cell behavior can be visualized dynamically *in vivo*. Zebrafish larvae are translucent and develop rapidly (24), and early skeletal processes can be dynamically visualized in the living fish through use of fluorescent transgenic reporter lines marking these cell types (see Table 1 for examples). Formation of the craniofacial skeleton occurs early, with the first cartilaginous structures of the jaw forming by 2 days post fertilization (dpf) (66), the first skeletal joints are formed and mobile by 3 dpf (60), by 5 dpf, hypertrophic chondrocytes, marked by *col10a1a*, are seen in some elements from 5 dpf (29), and first osteoblasts surrounding the cartilage and forming bone matrix by 7 dpf (67). The first intramembranous bones, such as the cleithrum, anterior notochord, and operculum, are visible in the craniofacial skeleton from 72 hpf (66). While skeletal development occurs early, true remodeling through the combined activity of osteoblasts and osteoclasts does not commence until the second week of development as osteoclasts (marked by Cathepsin-K (Ctsk) or TRAP) are not formed until day 10–12. Unlike mammals, mononucleated osteoclasts as well as multinucleated cells are present and actively resorb bone (65, 67).

**Figure 2:**
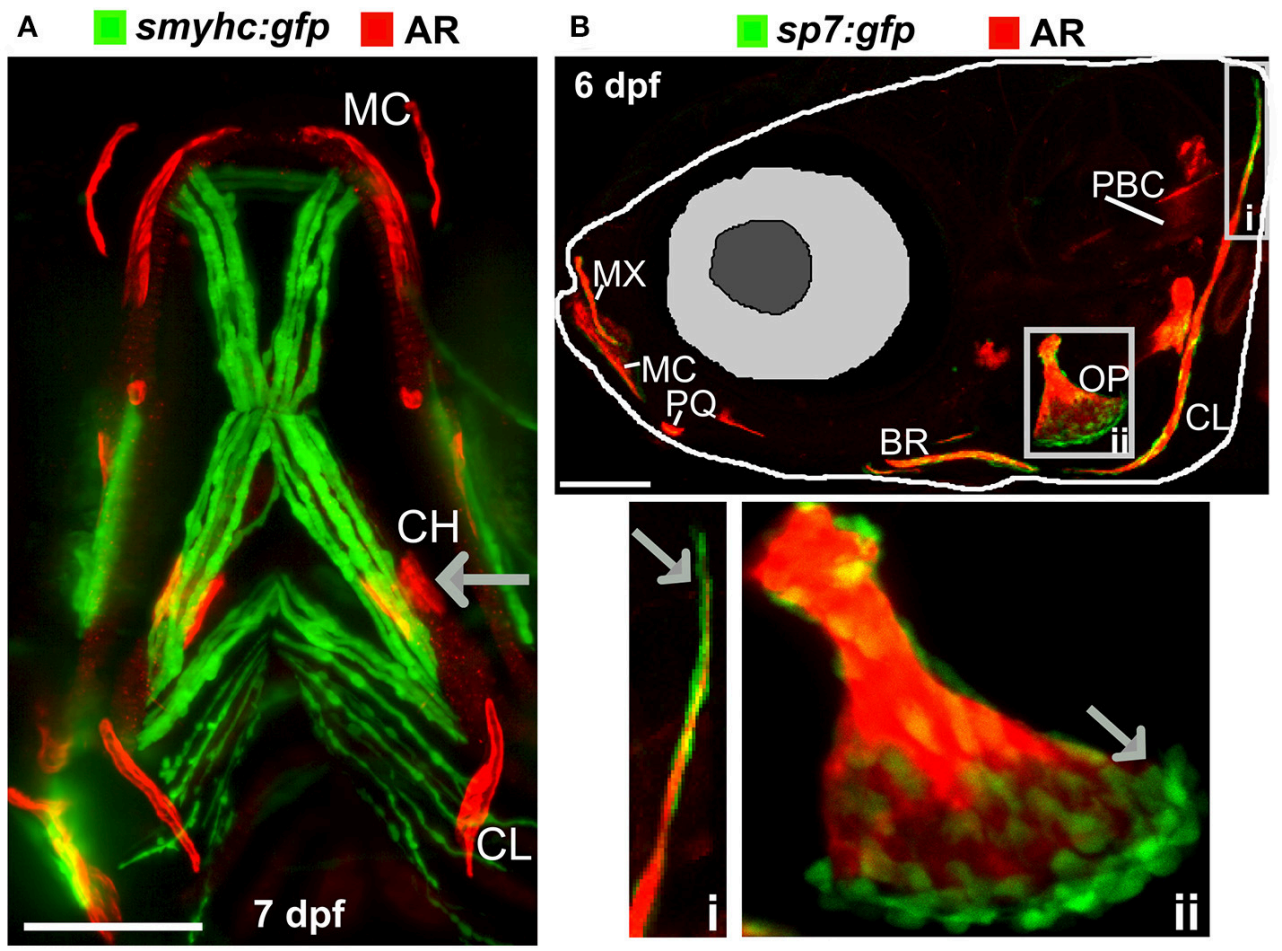
Ossified elements in the cranial region during early development. **(A)** Ventral view of a 7 days live Alizarin Red S (AR) labeled larval jaw showing dermal ossification of cleithrum (CL), and ossification of the cartilaginous ceratohyal (CH). Arrow indicates the CH which undergoes endochondral ossification. Slow muscle transgene reporter in green (*smych:gfp*). Image taken on a Leica lightsheet microscope. **(B)** Lateral view of a 6 days old larva live labeled with Alizarin Red S (red) and carrying GFP under the control of the osteoblast promoter s7/osterix (green; *sp7:gfp*) allowing visualization of mineralized elements (red) and osteoblasts (green) in a living individual. Insets show the cleithrum (i) and operculum (ii) with osteoblast enrichment at the distal ends of these elements (gray arrows). Image taken on a confocal microscope. Wildtype strains AB/TL in both panels. Ossified elements: BR, branchiostegal ray; CH, ceratohyal; CL, cleithrum; MC, Meckel's cartilage; MX, maxilla; OP, operculum; PBC, posterior basicranial commissure; PQ, palatoquadrate. Scale bars = 100 μm.

There are many transgenic lines available to mark musculoskeletal tissues, these include reporter lines which label cells or signaling pathway activation by driving expression of proteins in the cytoplasm, targeted to the nucleus, or plasma membrane, and lines that tag proteins (28, 68). Reporter lines mark cell types by using a tissue specific promotor, responsive elements from a signaling pathway, or transcription factor binding sites controlling expression of a fluorescent protein (Table 1). For example, to study bone homeostasis, osteoblasts and osteoclasts can both be labeled *in vivo*, using osteoblast reporters such as *sp7*, and osteoclast reporters such as *ctsk*, so that their numbers, location and activity monitored in living bone tissue either longitudinally, in response to drug treatment, genetic mutation, or environmental stimuli (Table 1). Relevant to research into OP, osteoclasts can be specifically temporally activated by use of a heat shock promoter driving RANK ligand (*rankl*) expression; such that following a period of immersion in water at 39°C, osteoclast activity, labeled with the blue fluorescent protein (CFP), is increased resulting degradation of the bone matrix and in an osteoporotic phenotype of low BMD (34). A simple Alizarin Red S (AR) staining, which marks calcium phosphate crystals and fluoresces strongly in the red channel (580 nm wavelength), allows a rapid assessment of ossified elements in live or fixed fish. In combination with transgenic lines, endochondral ossification in the lower jaw (Figure 2A) and intramembranous bone formation in the operculum (Figure 2B) can be easily visualized compared to traditional rodent models.

## Imaging the Adult Skeleton for Assessing Mineralization

**Figure 3:**
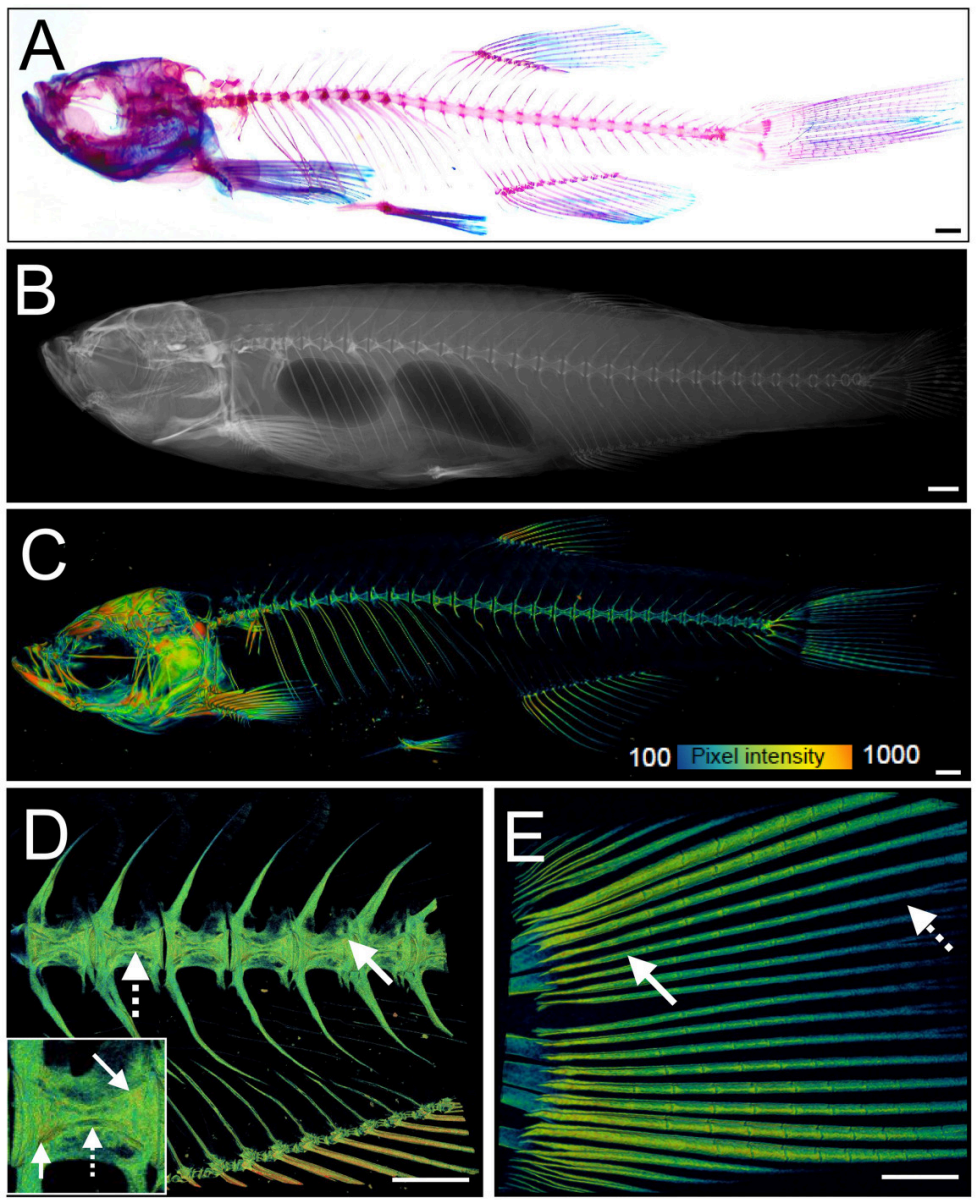
Examples of visualization and quantification of mineralized bone in zebrafish. **(A)** Wholemount Alizarin Red S (AR) and Alcian Blue staining of 3 months fixed fish. **(B)** Radiograph of 1-year old live fish showing whole body: endo- and exoskeleton. **(C)** Low resolution μCT images acquired with a 20 μm voxel size of a 3 months old fish. Note that pixel intensity can be used to determine BMD; represented on the color coded pixel intensity bar. **(D,E)** High resolution (5 μm voxel size) μCT images of vertebral column with anal fin rays **(D)** and caudal fin rays **(E)**. Vertebral centrae have higher density at their edges (solid arrow) than the center (dashed arrow). In the fin rays, a higher density (solid arrow) is observed in older segments within the proximity to the body in comparison to younger segments located more caudally showing lower pixel intensity (dashed arrow). The same pixel intensity color coding as **(C)** applies. All fish and their insets are depicted from a lateral view in an anterior-posterior (left-right) orientation. Scale bars = 50 μm in **(A,B)**; and 100 μm in **(C–E)**.

The zebrafish adult skeleton is relatively complex and once fully formed by around 2 months is composed of 74 ossified cranial elements (compared with 22 in humans), 28–31 vertebrae; 4 cervical, 10–11 thoracic vertebrae, and 15–16 separated vertebrae in the tail region and fins (pectoral, dorsal, anal (ventral), and caudal) (69). As in larvae, live AR and Calcein staining, or use of transgenic lines, allows easy detection of superficially located calcified elements in the skull, elasmoid scales, and fins using a simple fluorescent microscope. Deeper tissues can be imaged by multiphoton microscopy in small juveniles. However, bones located more internally (e.g., vertebrae and ribs) in large adults are difficult to visualize using this method. Post-mortem staining of bone (AR) and cartilage [Alcian blue (AB)] is a cost-effective way to analyse these structures for adult skeletal abnormalities (Figure 3A) and has been used in forward genetic screens to obtain detailed skeletal morphology information (56, 70, 71).

Recent advances in X-ray based imaging: radiographs, micro-computed tomography (μCT), and synchrotron equipped μCT technologies (SR-μCT), and their subsequent downstream imaging processing, opened avenues to assess the adult zebrafish skeleton. The major advantage of using these X-ray imaging techniques is that they are non-destructive and can be used in the intact fish, allowing the samples to be used for other purposes, such as histology. Radiographs give two-dimensional (2D) images of the zebrafish skeleton at relatively low resolution (Figure 3B), permitting the visualization of bone elements and a broad evaluation of changes in the skeleton, radiographs can be used to image live anesthetized fish permitting longitudinal analysis of the skeleton over time (70). Higher resolution (2 μm voxel size) and three-dimensional (3D) assessment of the zebrafish skeleton can be achieved by using μCT (Figures 3C–E). As fish bone, like that of mammals, is composed of hydroxyapatite crystals, quantification of BMD can be performed by comparison to phantoms, which are samples of known hydroxyapatite content (72). Additionally, treatment with agents to improve contrast such as silver nitrate (AgNO_3_) or iodine, allow detection of juvenile (less dense) bone and of soft tissues such as muscle and cartilage (72).

Very detailed data on bone micro-architecture can be achieved with SR-μCT (73, 74). This technique can yield a spatial resolution of 100 nanometres on tissue samples and visualize fine bone structures at a cellular level including the vasculature in mineralized bone, osteoclast resorption pits, and osteocyte lacunae (75). As the size and resolution of data sets increase, the bottleneck in the process is frequently data analysis. Commercially available software packages such as “boneJ” are tailored for CT data analysis, and recently open source user friendly software have become available to process μCT data from zebrafish scans. For example, the “FishCut” software processes whole-body μCT scan datasets and applies semi-automated analysis algorithms. The current version segments the axial skeleton, then generates values for the surface area of vertebrae and centrae, and calculates BMD and mineralized thickness in a semi-automated fashion (76).

## Zebrafish Mutants of Brittle or Thin Bones

An increasing number of zebrafish genetic mutants in skeletally relevant genes have been shown to recapitulate human bone disease. These have provided insight into the dynamic regulation of bone formation, mineralization, and remodeling. We have included a list of zebrafish skeletal mutants in Table 2. While there are currently few models for OP, there are various zebrafish mutant lines that accurately model human skeletal dysplasias, including collagenopathies and forms of osteogenesis imperfecta, which are characterized by brittle bones and frequent low-impact bone fractures. Autosomal dominant mutations *COL1A1* and *COL1A2* genes predominantly affect glycine-X-Y (Gly-X-Y) repeat domains that result in collagen α1(I) and α2(I) heterotrimer maturation defects (119), causing fragile bone matrix and insufficient mineralization (120). The Gly-X-Y mutations lead to impaired hydroxylation and defects in collagen maturation in the endoplasmic reticulum (ER), which is also conserved in zebrafish (121–123). The autosomal dominant *chihuahua* (*chi*) zebrafish mutant, was identified in a forward genetic screen using radiography (70). Linkage mapping identified a mutation causing a glycine to aspartate amino acid substitution in a conserved Gly-X-Y repeat of *col1a1a* (zebrafish *col1a1* is duplicated). Note that in contrast to mammals, zebrafish type-I collagen is constituted by three different α chains [α1 (*col1a1a*), α3 (*col1a1b*), α2 (*col1a2*)] due to duplication (124). *chi/*+ zebrafish display phenotypes resembling those seen in humans, including a shortened axial skeleton, with irregular radiodensity, uneven mineralization, and brittle bones that fracture easily (especially ribs). Transmission electron microscopy revealed that *chi/*+ fish show signs of ER stress (70). The ER trapping of insufficiently hydroxylated oligotrimerized α1(I)/α2(I)/α3(I) collagen leads to lower extra-cellular collagen maturity, abnormally shaped and thinner vertebrae bodies, areas of higher calcium content, different local mechanical properties, and reduced osteocyte number (84). Osteogenesis imperfecta has a broad disease spectrum in the clinic, and recent comparative studies of multiple mutant alleles for *col1a1a, col1a1b, col1a2*, and also *bmp1a* (described later) and *plod2* described a diversity of skeletal phenotypes (Table 2) with brittle bones as the common feature (85).

**Table 2:** Zebrafish mutants, transgene insertion mutants, and morphants showing altered skeletal mineralization.

**Human gene**	**Zebrafish name**	**BMD effect**	**Primary defect/effect**	**Fish modeling**	**Human skeletal phenotype**	**Citation**
*ABCC6*	*gräte*	+	ATP hydrolysis defects causing (ectopic) increased mineralization in spine and soft tissues	N/A	Pseudoxan-thoma elasticum	(77)
*ATP6V1H*	*atp6v1h*	–	Increased osteoclast activity by upregulated *mmp9* and *mmp13*	Osteoporosis	Familial osteoporosis with short stature	(78)
*BMP1*	*frilly fins, welded*	–	Fibrillar collagen processing affecting bone matrix integrity	Osteogenesis imperfecta	Osteogenesis imperfecta; high BMD (in vertebrae) but weak bones	(72, 79, 80)
*C-FMS (CSF1R/CD115)*	*panther, csfr1a*	+	Reduced osteoclast number and immune cell mobility causing stenosis	Osteopetrosis	N/A	(72, 81, 82)
*COL11A2*	*col11a2*	+	Collagen triple helical stability; dominant effect	OA: Stickler syndrome	Stickler Syndrome	(83)
*COL1A1*	*chihuahua, microwaved, dmh13, dmh14, dmh15, dmh29*	–	Collagen triple helix stability; dominant effect leading to brittle bones in axial and fin skeleton.	Osteogenesis imperfecta and Ehlers-Danlos syndrome (*chuhuahua* and *microwaved*)	Osteogenesis imperfecta and Ehlers-Danlos Syndrome	(56, 70, 79, 84–86)
*COL2A1*	*dmh21 (?), dmh27, dmh28, dmh30 (?)*	=	Collagen triple helical stability; dominant effect. Notochord and vertebra deformations.	Spinal deformations	Stickler syndrome	(56)
CTSK	ctsk ^*^^¥^)	+	Depletion of pre and mature osteoclasts	Osteopetrosis	Osteopetrosis	(87)
*CX43 (GJA1)*	*stoepsel, short-of-fin*	– (?)	Brittle vertebrae anomalies due to loss of function hemichannel (Ca^2+^) activity	N/A	Oculodento-digital dysplasia	(88, 89)
*CYP26B1*	*stocksteif, dolphin, cyp26b1*	+	Hyper-mineralization and fusion of the vertebrae and joints due to altered intracellular retonic acid metabolism	Retonic acid processing	Craniosynostosis, craniofacial anomalies, fusions of long bones	(37, 90, 91)
*DKK1 (DICKKOPF)*	hs:dkk^*^	–	When heat-shocked, Dkk1 is expressed and blocks Wnt/Beta-catenin signaling. Impaired elasmoid scale and ray fin outgrowth.	N/A	Osteolytic bone lesions in multiple myeloma patients	(92)
*EDA and EDAR*	*nackt (eda), finless (edar), fang (edar), topless (edar)*	–	Absence and deformation of dermal bone structures such as lepidotrichia, elasmoid scales, and skull	Ectodermal dysplasia, impaired teeth	Hypohidrotic ectodermal dysplasia 1 (X-linked); Tooth agenesis	(93)
*ENPP1*	*dragonfish*	+	Ectopic hyper-mineralization in axial skeleton due to altered phosphate metabolism	Arterial calcification of infancy	Arterial calcification /hypophosphatemic rickets	(94, 95)
*ENTPD5*	*no bone*	–	Does not mineralize bone due to altered phosphate metabolism	N/A	N/A	(94)
*GBA1*	*gba1*	–	Impaired osteoblast differentiation due to altered Wnt signaling	Osteoporosis, Gaucher disease	Osteoporosis, Gaucher disease	(96)
*GLI2*	hs:gli2-DR^*^	–	Heat-shock (hs) initiates expression of dominant repressive Gli2. Impaired scale calcification.	N/A	Culler-Jones syndrome; holoprosencephaly	(92)
*GOLGB1 (giantin)*	*golgb1*	+	Ectopic mineralization in spine and soft tissues by transcriptionally down regulating *galnt3* and changed cilia morphology	N/A	*GOLGB1* unknown–*GALNT3* mutations cause tumoral calcinosis	(49, 50)
*IHH*	*ihha*	–	Loss of mineralization due to blocked osteoblast differentiation in endochondral bone. Irregular operculum and scale morphology with reduced AR stain	Endochondral bone repair and dermal ossification	Acrocapitofemoral Dysplasia, Brachydactyly Type A1	(67, 97–99)
*ITGA10 ITGBL1 ^#^*	*itga10 ($) itgbl1 ($)*	–	Focal adhesion Integrin A/B subunits. Downregulated in prednisolone larvae.	Osteoporosis	N/A	(100)
*LGMN*	*lgmn ($)*	+	Legumain (secreted cysteine protease) inhibits osteoblast activity by degradation of fibronectin	Osteoporosis	Osteoporotic–upregulated in OP bone	(101)
*LRP4*	*lrp4 MO*	– (?)	Malformed pectoral and tail fin and deformed craniofacial skeleton with kidney cysts	Cenani-Lenz syndactyly	Cenani-Lenz syndactyly, osteoporosis, Sclerosteosis	(102)
*MEF2C*	*mef2ca*	+	Ectopic bone formation of neural crest derived ligament due to altered DNA methylation	N/A	Unknown	(103, 104)
*N/A*	*bone calcification slow*	–	Non-mapped mutation causing delayed ossification and increased Cyp26b1 expression	N/A	Unknown	(105)
*PANX3*	*panx3 MO*	–	Altered Ca2+ channel activity reducing endochondral ossification	N/A	N/A	(106)
*PLS3*	*pls3 MO*	–	Reduced larval operculum mineralization	Osteoporosis	X-linked osteoporosis	(107)
*PTCH1, PTCH2*	*ptch1 (ptc2), ptch2 (ptc1)*	+	Increased mineralization in endochondral bone	N/A	Holoprosencephaly	(67)
*PTH4 ^#^*	pth4^*^	–	Neuronal regulation of phosphate metabolism	N/A	PTH4 is absent in terrestrial animals	(108)
*PTHrP / PTHLH / PTH3*	*pthlha/pthlhb MOs*	+	Premature ossification during larval stage under control of sox9	N/A	Brachydactyly; mutation in promoter	(109)
*RANKL*	*rankl* ^¥^^*^)	–	Induces osteoclast activity	Osteoporosis	Osteoporosis	(34)
*RPZ ^#^*	*rapunzel*	+	Increased BMD in craniofacial and spinal column elements	N/A	None–Teleost specific gene	(110)
*SLC10A7*	*slc10a7 MO*	–	Secretory pathway defect	N/A	Decreased BMD; skeletal dysplasia	(111)
SP7 (OSX, osterix)	*sp7 (osx, osterix)*	–	Decreased mineralization, skull sutures defects, impaired teeth formation, increased BMP signaling, and reduced differentiation, but increased proliferation, of osteoblasts. Homozygous mutant adults are viable	Osteogenesis imperfecta, osteoporosis (?)	Osteogenesis imperfecta	(112, 113)
*SP7 (OSX, osterix)*	*sp7 (osx, osterix)* ^¥^)	–	Decreased mineralization of endochondral bone and vertebrae. Reduced osteoblast number. Homozygous lethal at 14 dpf	Osteogenesis imperfecta	Osteogenesis imperfecta	(114, 115)
*SPP1*	*spp1 (osteopontin)$*	–	Reduced AR staining in 5 dpf craniofacial skeleton. Absent in whale shark genome	N/A	N/A	(116)
*TGFB3*	*tgfb3 MO*	–	Reduced calcification of juvenile bone	N/A	Oral clefting	(117)
*TSHR*	*opallus*	+	Mutation causes a constitutive active Tshr leading to hyperthyroidism causing high BMD	Hyperthyroidism	Hyperthyroidism	(76)
*TWIST and TCF12*	*twist1b and tcf12*	+/=	Frontal skull sutures due to increased osteoblast proliferation. Mineralization normal.	Saethre-Chotzen syndrome	Saethre-Chotzen syndrome	(118)

$ *Mosaicism; MO, Morpholino*;

# *No clear ortholog*;

(?) *Indicated / implied*;

* *Transgene affecting gene*;

¥ *Medaka*.

The zebrafish *sp7/osterix* mutant has been shown to model human osteogenesis imperfecta caused by recessive damaging mutations in *SP7* (125). This mutant showed uneven mineralization, severe fractures caused by minimal impact, and misshapen bones. Moreover, rare craniofacial characteristics caused by impaired SP7 function, such as wormian bones, reported in human patients carrying mutations in *SP7* were also observed in zebrafish (112).

Another example of a zebrafish mutant that recapitulates patient phenotype is the *bmp1a* mutant *frilly fins* (*frf*). In humans a damaging missense mutation in the BMP1 signal peptide causes brittle bones in an osteogenesis imperfecta pedigree (79). *frf* mutants showed normal osteoblast number, but pericellular pro-collagen processing (C-pro-peptide removal) defect leading to mineralization defects in the axial skeleton and fin rays (79).

Collagenopathies, such as Stickler Syndrome, have also been successfully modeled in zebrafish. We have recently reported a *col11a2* zebrafish mutant showing specific traits of the human disease which include thicker collagen fibers and degradation of type-II collagen in zebrafish larvae leading to compromised jaw shape, mechanical properties and movement of the jaw leading to premature OA (83). In many skeletal dysplasias zebrafish not only model the human condition but allow mechanistic insight into how genetic changes lead to the cellular changes that underpin the disease symptoms. As such zebrafish offer exciting prospects for delivering functional studies in new osteoporotic genetic loci.

## Assays of Caudal Fin Regeneration and Fracture Repair to Asses *de novo* Bone Matrix Formation

**Figure 4:**
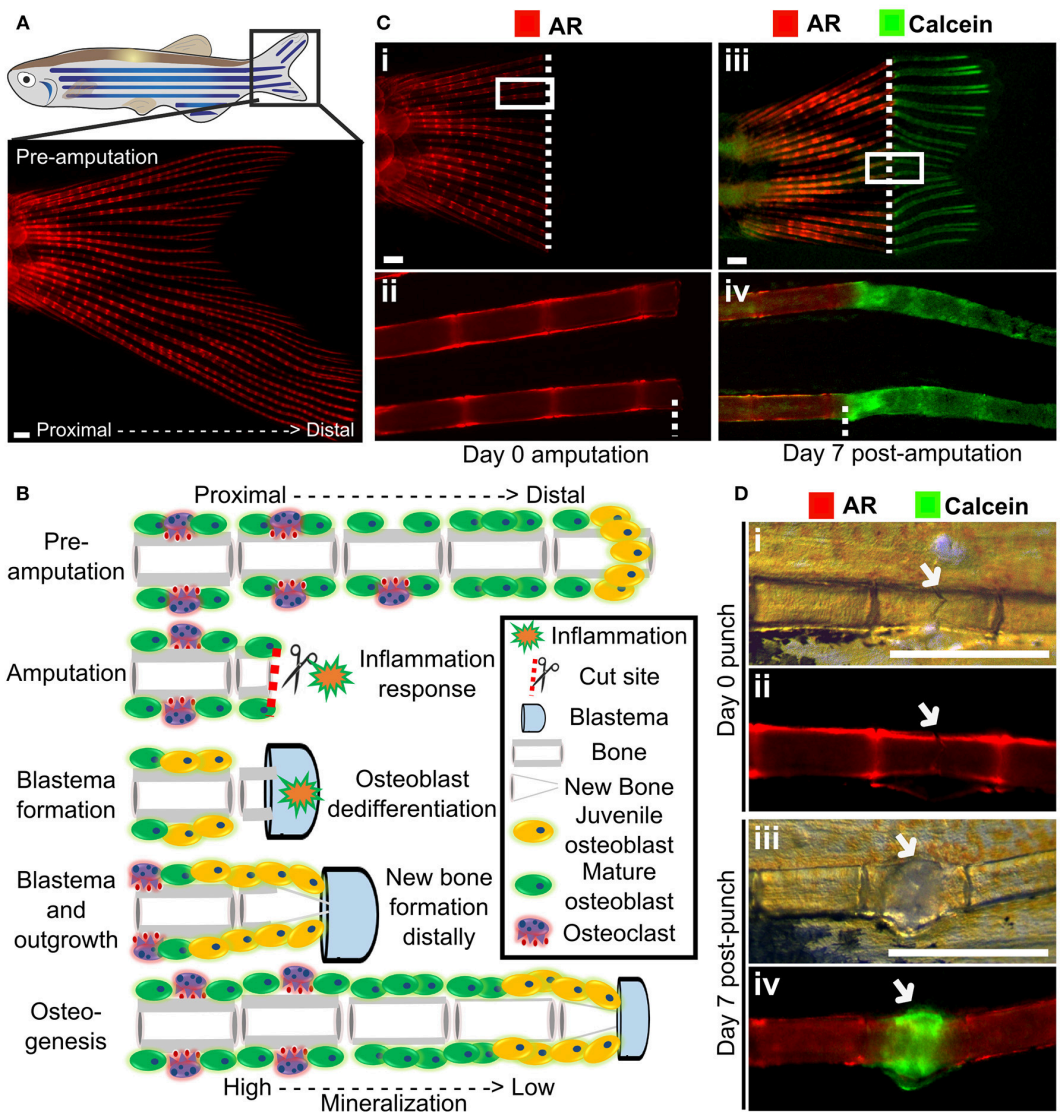
Fin regeneration and fracture assay to visualize and quantify live bone formation and repair. **(A)** Schematic representation of a zebrafish with a standard fluorescent stereomicroscope image of a live Alizarin red S (AR) pre-amputation caudal fin (inset). **(B)** Schematic representation of bone regeneration after fin amputation showing the (simplified) cascade of events that follow after fin amputation to regenerate bone (a single ray depicted here). This allows studying *de novo* bone formation by newly formed osteoblasts (orange cells) and differentiated osteoblasts (green cells) and subsequent remodeling by osteoblasts and osteoclasts (purple cells) in an adult fish. Note that during osteogenesis that there is a gradient of mineralization. **(C)** Live images of the tail fin labeled with Alizarin red (red) prior to amputation (i, ii) and Calcein (green) post-amputation (iii, iv) taken on a fluorescent dissecting microscope. All images in panel come from the same fish. Seven days post-amputation showing regrowth of new bone (green). Note that intense Calcein staining is visible distally from the amputation site (white dotted line). **(D)** The fracture healing assay involves applying pressure on a fin ray bone element to induce a small fracture to one segment of the fin ray (i), which is visible with life AR staining (ii). Green Calcein labels the new bone formed in the fracture callus by 7 days (iii and iv). The white arrow indicates the fracture site. Scale bars = 500 μm, 3 months old wildtype TL/EKK females.

Zebrafish are capable of regeneration many tissues and organs including the heart, lens, and pancreas. They also show regeneration of skeletal tissues following amputation of the tail fin (lepidotrichia) or removal of elasmoid scales (126, 127). As the fins and scales are translucent, and readily imaged they allow cells and their calcified matrix to be visualized in detail using standard fluorescent microscopes (Figure 4A). After amputation of a ray fin (typically a caudal fin), a wound healing response results in the formation of an epimorphic blastema which regenerates all affected tissues of the amputated organ, including bone, in a controlled fashion (128). Following this inflammation response, osteoblasts undergo dedifferentiation and proliferate to contribute to the blastema (33, 129). These juvenile osteoblasts then secrete matrix with intermediate properties between cartilage and bone and are later remodeled as mature bone by matured osteoblasts and recruited osteoclasts (Figure 4B) (33, 128). These fins can also be injured via cryo-injury by placing a −196°C knife perpendicularly to the caudal fin rays allowing to study the bone resorption response (130). These techniques offer great perspectives to compare bone formation and bone remodeling, in an adult context.

Using transgenic lines and *in vivo* staining methods, such as AR (fluoresces red, 545 nm excitation, 580 nm emission) and Calcein (fluoresces green, 495 nm excitation, 515 nm emission), which binds to calcified matrix, the dynamics of bone formation can be visualized by using a fluorescent stereomicroscope in a regenerating caudal fin of a living fish. This allows longitudinal analysis by following regeneration rate and volume, since AR stains fully mineralized bone and Calcein binds to newly deposited bone matrix (Figure 4C).

The utility of fin regeneration assays to test bioactive compounds has been demonstrated by treating regenerating fins with the glucocorticoid prednisolone. Following treatment bone formation was reduced, and furthermore, both osteoblast number and subsequent bone deposition and osteoclast recruitment was reduced in these fins (131). Interestingly, skull injury repair is less affected following prednisolone treatment (131), this is similar to mammals. Treatment of fins with *Botulinum toxin* (Botox) leads to a reduction in bone mineralization and regeneration following amputation (132), comparable to the situation in mammals where fracture repair is impaired following Botox induced paralysis (133, 134).

A major issue with OP is increased fracture risk due to weaker bone structure, and therefore identification of therapeutics that can improve fracture healing is desirable. Zebrafish show a fracture healing response, including callus formation (Figure 4D), with strong similarities to that of mammals. Fractures can be induced in zebrafish fins using simple pressure applied externally to the fin (131, 135). As the fin has around 300 bony rays, multiple fractures can be induced in a single fin. A fracture callus is formed and *de novo* bone formation is initiated 2 days post-injury accompanied by an increased expression of osteoblast genes such as *runx2* and *sp7/osx* (131, 135). As the fin is flat, the fracture repair process can be dynamically tracked at cellular resolution using transgenic lines (Table 1) or by labeling bone formation with AR and Calcein (Figure 4D). As for regeneration, it is possible to add pharmacological agents to the regenerating tissue (131), allowing potential osteoanabolic compounds to be tested for beneficial effects in fracture repair *in vivo* (136).

## Skeletal Assays Using Elasmoid Scales

**Figure 5:**
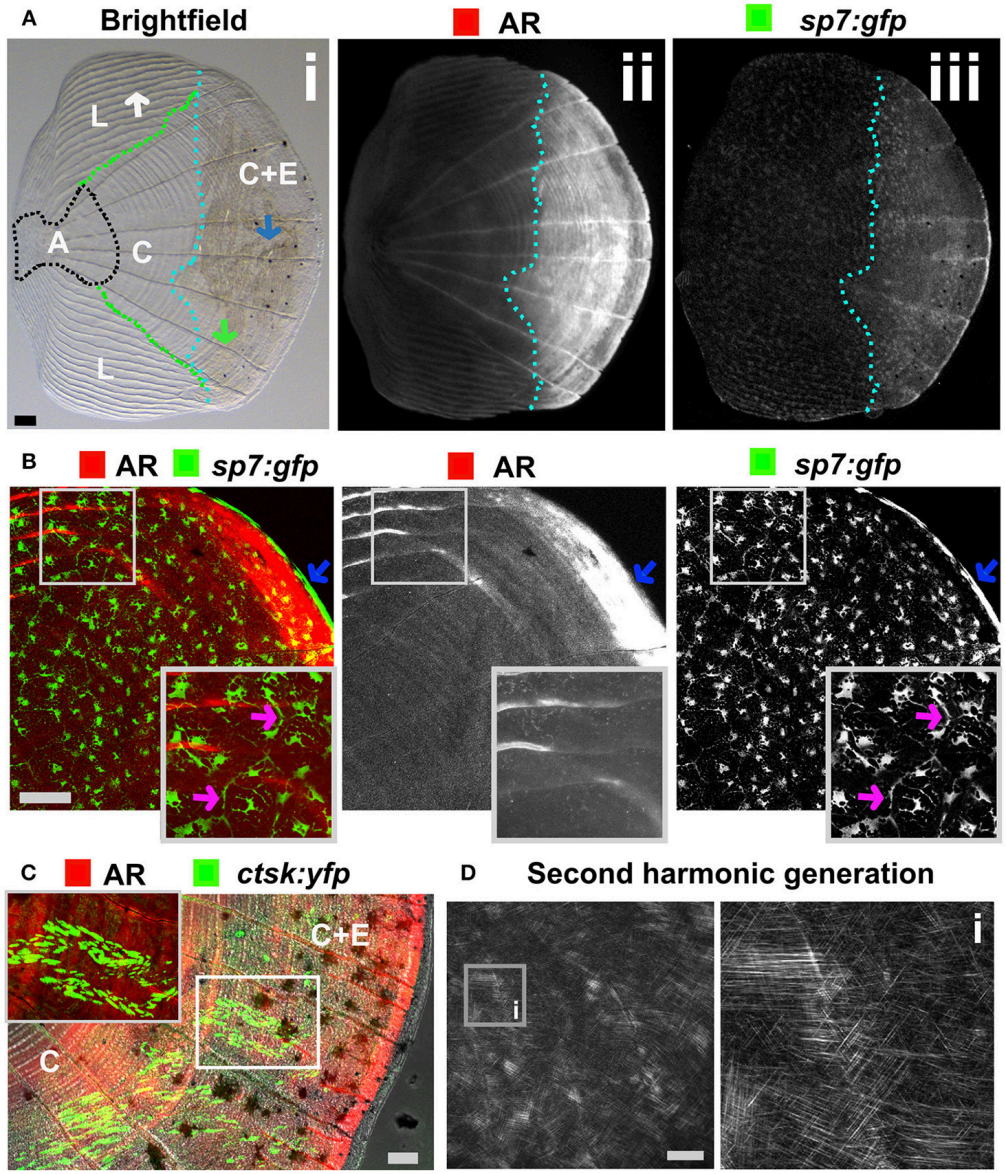
Zebrafish elasmoid scale structure and bone cell types. **(A)** Single scale from the flank of a 3 months old fish carrying the *sp7:gfp* osteoblast reporter transgene (green) and stained for Alizarin Red S (AR, red). Whole scale is shown in bright field (i) and gray scale images for AR (ii) and GFP (iii) in the top panels. The brightfield image (i) depicts the anterior anchor region (A, black dotted line boundaries), the lateral circuli (L, green dotted line boundaries, white arrow), central region (C, surrounded by black, green, and light blue dotted lines), and central region covered by epidermis (C+E, light blue dotted line, with grooves by green arrow) with enhanced mineralization. **(B)** Confocal images showing a merge image of osteoblasts (*sp7:gfp* transgenic fish, green) abundantly distributed over the freshly harvested scale and AR staining (red). Individual channels are depicted in gray scale images. Note increased mineralization at the edge of the scale corresponding increased GFP presence (blue arrows). Insets focus on the lateral circulus and note osteoblast cytoplasmic protrusions (pink arrows). **(C)** Confocal images visualizing osteoclasts with cathepsin K (ctsk) YFP reporter expression (green), mineralization by AR (red), and brightfield (gray). Note that YFP positive cells were predominantly seen in the central region with epidermis (C+E) and distal edges of the central region **(C)**. **(D)** Multiphoton forward scattering (second harmonic generation (SHG), 880 nm wavelength) visualizes collagen fibrils in an ethanol fixed scale. Inset (i) shows the organization of collagen fibrils in a plywood structure. Wildtype strains (panel): TL/EKK **(A)**, TL **(B)**, AB/TL **(C)**. Scale bars 100 μm.

The body of zebrafish is covered with elasmoid scales made of calcified dermal bone harboring osteoblasts and osteoclasts (Figures 5A–C). The calcified matrix is composed of a plywood structure of collagen fibrils (137), which are easily visualized with second harmonics generation microscopy (Figure 5D). Scales are embedded in, and grow from, the dermis and shed and replace naturally throughout life of the fish (138). As scales are part of the exoskeleton they are easy to collect from an anesthetized fish. Each flat scale is subdivided in four regions by its morphology: anterior, lateral, central, and central with epidermis (Figure 5A) (139). The anterior region is attached to the skin and does not grow or form new bone. The lateral area is characterized by its curved ridges (circuli), whereas the central area has linear trenches. Within the lateral circuli and central grooves newly mineralized matrix is formed by osteoblasts (139) and degraded by osteoclasts (140). The posterior area has increased osteoblast number and bone is continuously deposited (Figures 5A,B). Osteoblasts in different regions of the scale express different markers of maturity (97). As the scale contains living cells, including nerve and vascular endothelial cells, their use offers an opportunity to study bone cell behavior in a mature context.

## Pharmacological Manipulation of Bone Tissue and Chemical Genetic Screening

As larvae are small and develop in water, it is possible to grow larvae in multi-well format with the addition of water-soluble compounds to their growth media for easy uptake. Zebrafish have been used extensively for high-throughput screening using larvae and now drugs are used in clinical studies that were first identified in zebrafish. A great example is the identification of the kinase inhibitor dorsomorphin (BMP type-1 receptor (BMP1R) antagonist) to treat lymphoma which was discovered in an early embryogenesis phenotype screen using 7,500 small-molecules (141, 142). Another example used semi-automated imaging strategy of Calcein stained larvae exposed to a small-compound library identifying 6 catabolic and 2 anabolic compounds that alter notochord mineralization (143) (Table 3). Thus, when fluorescent compounds are twinned with fluorescent reporters for osteoblasts (e.g., *sp7:gfp* with AR) (Figure 2B), it will allow assessment of osteoblast number and activity in a semi-high content setting using plate imaging microscopy (162). When these assays are combined with high efficiency CRISPR/Cas9 genome engineering strategies, it will open avenues to test compounds of interest that could alter disease causing mutations deteriorating effects. Thus, this comprehensive approach will also offer opportunities to develop compounds for personalized medicine. For OP research it may be more advantageous to focus on adult skeletal assays to allow assessment of osteoclast activity (bone catabolism) simultaneously with an assessment of osteoblasts (bone anabolism). An example of pharmaco-genetics improving brittle bones, is when type-I collagen secretion in the bone matrix is ameliorated by treating *chi/*+ mutants with 4 phenyl butyrate (4PBA) compound (86).

**Table 3:** List of compounds, diets, and exercise that alter ossification in zebrafish larvae, adults, and/or adult elasmoid scales.

**Treatment**	**Gene/pathway**	**BMD effect**	**Primary effect**	**Part of compound screen?**	**Life stage**	**Citation**
4PBA	HSP47–ER protein/fibrillar collagen folding	+	Increased mineralization in both WT and chi/+ fish due to better clearing of type-I collagen from ER	No	Adult larval	(86)
Alendronate / etidronate	Alendronate/etidronate therapies (bisphosphonates)	+	Counteracts the negative effects of GIOP on scales. Reduced TRAP and increased AL activities.	No	Adult larval	(144, 145)
BGJ398	FGF-receptor kinase inhibitor	–	Reduced sp7 positive osteoblasts in elasmoid scales resulting in impaired scale growth	No	Adult	(92)
BML-2832 library	Alkaline phosphatase inhibitors	+/–	Six catabolic and two anabolic compounds affect larval mineralization of the vertebral region.	Yes	Larval	(143)
BMP-2a	BMP pathway	+	Increased *sp7:luciferase* activity on cultured scales	No	Adult	(38)
Botulinum toxin	Botox muscle paralyzes	–	Lower BMD and bone deposition in fin ray bones due to muscle paralysis. Impaired osteoblast differentiation.	No	Adult	(132)
Cobalt chloride	Down-regulation of stem cell markers	–	Reduced number of osteoblasts and subsequent mineralization of the operculum, without affecting its size.	No	Larval	(146)
Cyclopamine and BMS-833923	Hedgehog pathway	–	Smaller scales and fins during regeneration. Scales show a lower number of osteoblasts.	No	Adult	(97, 147)
Dexamethasone	Glucocorticoids	–	Glucocorticoid pathway inducing osteoporosis (GIOP) by inhibiting osteoblast activity	No	Adult larval	(148)
DMP-PYT	BMPII-R–SMAD1/5/9	+	Increased BMP (pSMAD1/5/8(9)) and WNT signaling in 6–7 dpf larvae exposed for 4 days.	Yes, C2C12 cells	Larval	(149)
Dorsomorphin	BMPI-R–SMAD1/5/9	–	Reduced BMP (pSMAD1/5/8(9)) and ALK activity, reducing osteogenesis by inhibiting osteoblast activity.	Yes, compound libraries	Embryo Larval	(141)
Ferric ammonium citrate	Radical Oxygen Species	–	Iron overload down regulating osteogenic markers which can be rescued with *hepcidin1* overexpression	No	Adult larval	(150, 151)
High fat diet	Obesity risk factor for OP	–	Increased osteoclast activity in elasmoid scales	No	Adult	(152)
High glucose diet	Hyperglycemia OP risk factor	–	Increased osteoclast activity and peripheral bone degradation in elasmoid scales	No	Adult	(153)
Hyper-gravity	Increased loading	+	Enhanced mineralization after exposure to 3 g in a large diameter centrifuge	No	Larval	(154)
Niclosamide, Riluzole, Genistein	WNT pathway	+	Increased *sp7:luciferase* activity on cultured scales	Yes, WNT compound library	Adult	(38)
N-LLEL and anandamide	Long-chain fatty acids binding cannabinoid type receptors	+	Higher alkaline phosphatase activity and protecting effect on the alteration of bone markers induced by GIOP	Yes, on scales	Adult	(155)
Oligosaccharides	*A. bidentata* oligosaccharides	+	Dried root extract of Asian medicinal herb reducing osteoclast and increasing osteoblast activities	No	Larval	(156)
Omega-6 Arachidonic acid	Omega-6 derivative	–	Stimulating matrix metalloproteinase activity Enhanced bone turnover by increased osteoclast activity in the scale.	No	Adult	(157)
Prednisolone	Glucocorticoids	–	Glucocorticoid pathway inducing osteoporosis by inhibiting osteoblast activity	Yes, used as OP control	Adult larval	(100, 140)
R115866	Cyp26 antagonist–retonic acid metabolism	+	Hyper-mineralization of axial skeleton and phenocopying of *stockteif* mutant phenotype	No	Larval	(37)
Retonic acid	Cyp26b1 and collagen deposition	+	Altered collagen deposition due to increased activity of Cyp26b1	No	Larval	(37, 158)
RU486	Glucocorticoid receptor antagonist	+	Used as prednisolone specificity/toxicity control–reverses its catabolic effect	No	Larval	(145)
SD-134	Inhibits legumain (LGMN) protease domain	+	Increase in larval vertebrae mineralization after 4 days of exposure (7 dpf)	No	Larval	(101)
Sodium metasilicate	Silicate ion	+	Silicate ion stimulating osteoblast function	No	Larval	(159)
SU5402	FGF-1 receptor antagonist	–	Impaired osteoblast proliferation in amputated fins	No	Adult	(33)
Swimming exercise	Bone loading	+	Zebrafish performed controlled exercise in a tunnel have a higher vertebrae BMD compared to non-exercising fish	No	Adult	(160)
Tanshinol	D(þ)b-3,4-dihydroxyphenyl lactic acid	+	Herbal extract reducing oxidative stress and reduction of glucocorticoid induced osteoporosis phenotype.	No	Larval	(148)
Teriparatide	Teriparatide (parathyroid hormone)	+	Human osteoporosis treatment increases mineralization in GIOP fish.	No	Larval	(161)
Vitamin D3	Cholecalciferol and calcitriol	+	Enhanced mineralization in prechordal sheet and cleithrum due to altered calcium uptake.	No	Larval	(146, 161)

Zebrafish elasmoid scales are bony plates that are small and contain bioactive osteoblasts and osteoclasts (Figures 5B,C). These therefore offer huge potential as a primary pharmacological screening tool for skeletal compounds. The scales can be cultured for 72-h post-harvesting during which they can arrayed in multi-well plates and exposed to pharmacological compounds. As the scale is thin, osteoblasts are accessible to osteogenic factors, and have been demonstrated to react in a dose dependent manner to BMP-2 (38). To model an OP-like phenotype, individuals can easily be exposed to prednisolone / dexamethasone (glucocorticoid pathway) (140, 148), ferric ammonium citrate (150, 151), or metabolically with a high fat or glucose diet (152, 153), see also Table 3. In the context of glucocorticoid induced OP (GIOP), the bisphosphonate Alendronate reverses the effects of prednisolone on *ex vivo* cultured elasmoid scale bone, which showed a reduction in osteoclast activity (measured by TRAP) and an increase in bone anabolism (measured by alkaline phosphatase activity) (144); the same response as in mammals (163, 164). As fat metabolism has been implicated with OP, a small fatty acid derivative library was used on GIOP adult fish. Biochemical assays on scales derived from these fish showed that cannaboid receptor 2 binding anandamide and N-linoleoylethanolamine (N-LLEL) fatty acids drive osteogenesis by stimulating alkaline phosphatase (ALK) activity (155).

**Figure 6:**
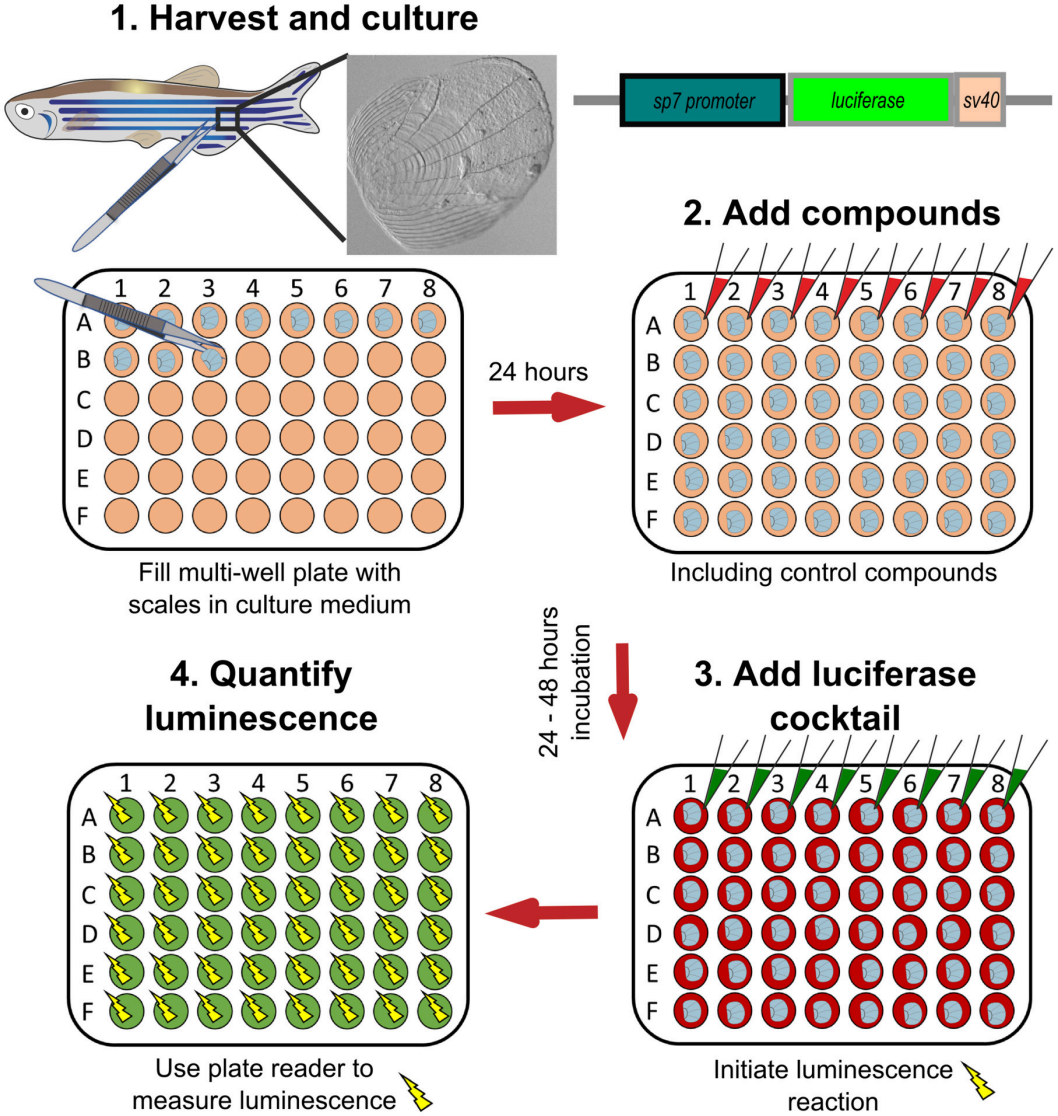
Schematic representation to show how osteoblast activity can be quantified from scales. Scales from *sp7:luciferase* transgenic reporter fish are harvested from the lateral flanks of a fish, then cultured in multi-well plates with DMEM culture medium (orange wells) at 28°C for 24 h. Compounds of interest can then be added (red wells) to the scales and incubated prior addition of a luciferin cocktail (green wells) and measurement of luciferase activity with a luminescent (yellow sparks) plate reader. Based on text from de Vrieze et al. (38).

A WNT-pathway compound library was tested to identify new osteo-anabolic compounds using an assay in which luciferase was expressed under control of the *sp7* promoter allowing a quantitative readout of osteoblast activity (Figure 6). This screen identified three osteo-anabolic (Table 3) and 15 osteo-catabolic compounds from 85 trial compounds (38). This library contained five previously published compounds tested *in vivo*, and nine tested *in vitro* mammalian bone progenitor cell lines. Strikingly, this scale luciferase assay was able to reproduce the effect of all *in vivo* tested compounds and about half of all *in vitro* tested compounds (38). These studies demonstrate the exciting potential that scale assays represent for testing of skeletal compounds relevant to OP in a cost-effective manner.

## Potential Drug Discovery Pipeline for Osteoporosis

**Figure 7:**
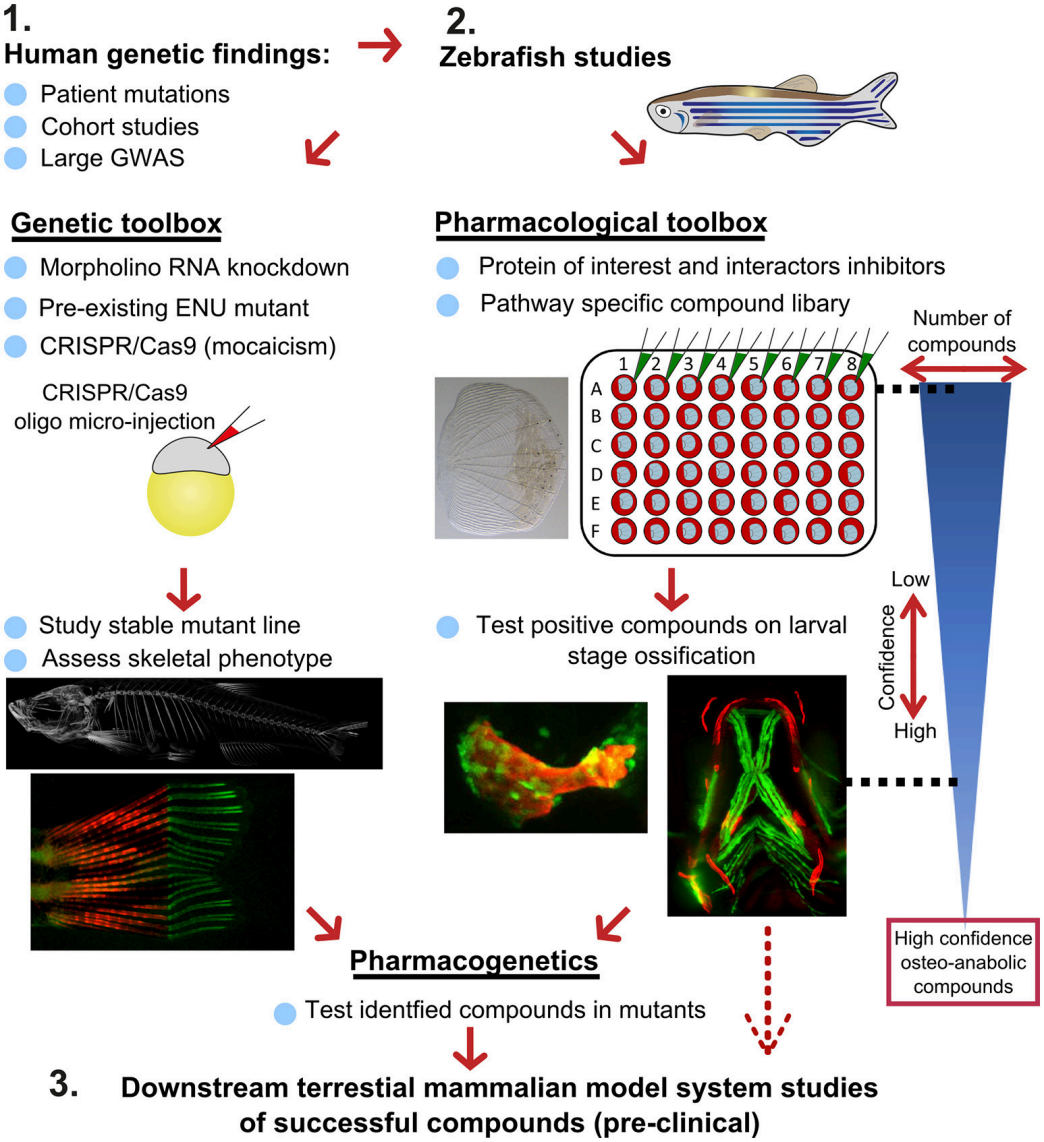
Proposed pipeline using zebrafish as a primary testing platform to address bottleneck for fast and affordable translation of human genetic findings. Two experimental arms using the genetic and pharmacological toolboxes allow simultaneous drug target validation. The blue reversed triangle depicts the reduction in number of putative osteo-anabolic compounds (along with an increase in confidence) when testing the compounds using the skeletal assays available.

Recently, there has been a substantial expansion in the quantity of high-quality genetic data from large-scale human genomic and transcriptomic studies that contain potential osteo-anabolic factors. Here we describe a potential screening pipeline that makes use of the genetic tractability and imaging in zebrafish to offer a relatively low cost, high-throughput option compared to traditional *in vitro* and *in vivo* models (Figure 7).

After identification of several candidate genes/drug targets from human genetic studies, the pipeline consists of two experimental arms that can be carried out simultaneously to generate primary pre-clinical data to validate the putative drug targets. Using genome editing, loss-of function studies can be performed in transgenic backgrounds to test the effect of the gene of interest on the developing skeleton or on mineralization, and simultaneously allowing safety testing for deleterious effects on other tissues or organs. For example, using CRISPR/Cas9 editing, it is possible to generate hundreds of mosaic zebrafish mutants within 3–4 weeks (includes the generation of the targeting reagents), which is difficult to achieve in other available systems, such as cultured chondrocytes and osteoblasts (differentiation of these takes multiple weeks). With CRISPR/Cas9 editing it is also feasible to study the specific human disease mutation in zebrafish, as long as it is in a conserved coding region. These fish can be grown to adulthood and germline mutations identified allowing more detailed studies on the mature skeleton to be performed (Figure 7).

In addition to genetic studies, pharmacological assessment of the identified putative drug target can be performed. By using water-soluble compounds, or lipid soluble compounds dissolved in DMSO, screens in a multi-well format can be performed using *ex vivo* culture of elasmoid scales. As a single adult fish has around 200 scales (138), this assay allows testing of many compounds, including control compounds (e.g., osteo-anabolic (alendronate) and -catabolic (prednisolone), on scales harvested from a single individual, reducing intra-individual variation (38). Therefore, this technique offers a platform to generate a primary read-out of novel osteo-active compounds in the context of homeostasis in a mature tissue. Additionally, this *ex vivo* technique will reduce the number of (potentially harmful) compounds being exposed to living fish, therefore contributing to ethical refinement and reduction of experimental animal use, but also reducing associated costs. As this scale assay reduces the number of putative osteogenic compounds substantially, these positive compounds can be further validated (along with safety testing) on developing transgenic larvae. These larvae would be plated out at 3 larvae per well and the compounds added from 3 days of development, with high-content imaging used for preliminary assessment of the effects of each compound and more detailed analysis including dose response followed up for validated positive hits. Further downstream tests, such as fin regeneration or fracture assays, can further reduce the number of compounds as such that only high-confidence compounds will be assessed in tetrapod pre-clinical studies (Figure 7).

If desired, the two experimental arms can be performed simultaneously, so that stable mutants are being generated during the compound testing phase. This opens the possibility to perform pharmacogenetic experiments in a relatively short time frame to validate the effects of putative drugs on specific disease mutations to see if they can “rescue” the disease phenotype (Figure 7). Together, zebrafish offer the potential in future to bridge the gap between human genetic hits, and fast functional validation.

## Prospects for Zebrafish in Osteoporosis Research

The zebrafish is a well-established increasingly used animal model for studying various diseases including (congenital) metabolic bone diseases (165). As zebrafish have historically been mainly used for its fast-embryonic development properties to better understand disease onset, zebrafish aging studies have only recently been conducted to model age-related diseases such as OA and OP. OP is an emerging field in zebrafish modeling and more research is needed to fully establish an OP-like phenotype as it was previously determined in its teleost cousin medaka (34). The advantageous properties as set-out in this review should be further exploited to benefit drug development for OP. Zebrafish show the appropriate response to increased mechanical loading, where the cellular (transcriptional) response initiates increased bone formation and mineralization in the loaded bone elements that are easily quantified (154, 160). However, since zebrafish and mammalian bone morphology show some differences (64), a pharmacological assay should particularly focus on the complex tissue and osteoblast-osteoclast interactions that underpin OP pathology. As traditional rodent and *in vitro* co-culture both have limitations to pursue large-scale drug discovery in a genetic context, zebrafish can take the place as a primary testing platform and therefore opening avenues to work toward gene specific compound discovery that have been identified as risk factors in human genetic studies. After primary safety testing, these identified compounds can be further tested in mammalian OP models to determine the effect on BMD, bone strength, and trabeculation. Fully exploiting these opportunities by using zebrafish as a primary screening model will open exciting avenues to perform pharmacogenetics for OP on a larger scale.

## Ethics Statement

Zebrafish procedures were approved by the University of Bristol Animal Welfare and Ethical Review Body (AWERB) and performed in accordance with a UK Home Office project license.

## Author Contributions

DB and EK generated the figures for the manuscript. DB, EK, and CH researched and drafted the manuscript together.

### Conflict of Interest Statement

The authors declare that the research was conducted in the absence of any commercial or financial relationships that could be construed as a potential conflict of interest.
